# A >200 Wh kg^−1^ anode-free Na pouch battery at −40°C enabled by manipulating electrolyte equilibrium

**DOI:** 10.1093/nsr/nwaf124

**Published:** 2025-03-29

**Authors:** Qiaonan Zhu, Jiawei Wang, Liqiang Wu, Hao Lan, Jiangchun Chen, Liwei Cheng, Bin Zhou, Daojun Yang, Jie Yang, Mirtemir Kurbanov, Shuai Dong, Hua Wang

**Affiliations:** School of Chemistry, Beihang University, Beijing 100191, China; School of Materials and Chemistry, University of Shanghai for Science and Technology, Shanghai 200093, China; Beijing Xibei Power Technology Co., Ltd., Beijing 102600, China; School of Chemistry, Beihang University, Beijing 100191, China; School of Chemistry, Beihang University, Beijing 100191, China; School of Chemistry, Beihang University, Beijing 100191, China; Beijing Xibei Power Technology Co., Ltd., Beijing 102600, China; Beijing Xibei Power Technology Co., Ltd., Beijing 102600, China; Hydrogen Energy Research Center, PetroChina Petrochemical Research Institute, Beijing 100083, China; Arifov Institute of Ion-Plasma and Laser Technologies, Academy of Sciences of the Republic of Uzbekistan, Tashkent 100077, Uzbekistan; School of Chemistry, Beihang University, Beijing 100191, China; School of Chemistry and Chemical Engineering, Henan Key Laboratory of Biomolecular Recognition and Sensing, Henan D&A Engineering Center of Advanced Battery Materials, Shangqiu Normal University, Shangqiu 476000, China; School of Chemistry, Beihang University, Beijing 100191, China

**Keywords:** anode free, electrolyte, high energy density, low temperature, sodium batteries

## Abstract

Cryogenic energy-dense batteries are essential for cold-climate applications. Anode-free configuration raises great opportunities for maximizing the cell-level energy density of batteries. However, high energy density of low-temperature anode-free batteries is still plagued by the lack of competent electrolytes with combined high compatibility towards metal anode and stability at high voltages. Herein, we report a high-energy-density anode-free Na battery at low temperatures via electrolyte association-dissociation equilibrium regulation. The optimized equilibrium facilitates the desolvation process and the formation of inorganic-rich anode/cathode-electrolyte interphases, thus simultaneously promoting low-temperature kinetics and high-voltage stability of the battery. Consequently, a high Coulombic efficiency (99.90%) of Na plating/stripping and a wide electrochemical stability window of electrolyte (>4.3 V) at −40°C are achieved. The anode-free Al@C||NaNi_1/3_Fe_1/3_Mn_1/3_O_2_ pouch cell delivers a record-high energy density of 204 Wh kg^−1^_entire cell_ at −40°C among the reported low-temperature rechargeable batteries. This work represents a defining step for energy-dense batteries which can operate at low temperatures.

## Teaser

A >200 Wh/kg low-temperature anode-free Na battery is successfully fabricated by electrolyte chemistry regulation, which manipulates the electrolyte association-dissociation equilibrium to simultaneously promote the low-temperature kinetics and high-voltage stability of the battery.

## INTRODUCTION

Cryogenic energy-dense batteries are of great significance in meeting energy-storage requirements under harsh conditions [1–4]. Anode-free configuration, which is constructed with a bare current collector on the anode side, is expected to enable low-temperature batteries with high energy density [5–7]. Nevertheless, achieving anode-free batteries at low temperatures is still challenged due to the insufficient Coulombic efficiency (CE) of metal plating/stripping, which can be attributed to the readily formed ‘dead metal’, severe dendrite growth, and fragile solid-electrolyte interphase (SEI) layer under cryogenic conditions [8–11]. Up to now, only two pioneer works have reported the successful fabrication of low-temperature anode-free Na batteries [6,12]. However, restricted by the narrow electrochemical stability window of their electrolytes, the full potential of anode-free batteries remains unexploited, as they fail to take advantage of energy-dense cathodes with high cut-off voltages [13–16].

To achieve high-energy-density anode-free batteries for cryogenic operation, the following targets should be simultaneously satisfied for the adopted electrolyte: (i) high ionic conductivity, (ii) wide electrochemical stability window, and (iii) enabling metal plating/stripping with ultrahigh CE (>99.5%) at low temperatures [17]. Several electrolyte design strategies have been proposed in an attempt to satisfy these demands. On the anode side, utilizing associated salts or weakly coordinated solvents to reduce the desolvation energy and constructing inorganic-rich SEI is effective in achieving high CE at sub-zero conditions [12,18,19]. On the cathode side, electrolytes with contact-ion-pair (CIP)-rich solvation structure were reported to be resistant to decomposition at high voltage [20], because the CIP-rich structure can not only expels the readily oxidized free solvents from the cathode surface, but also facilitates the formation of anion-derived robust cathode-electrolyte interphase (CEI) film [21]. These strategies are beneficial for enhancing the low-temperature stability or energy density of alkali-metal batteries. However, it remains challenging to synchronously meet the requirements of ultrahigh CE and high voltage stability for anode-free configuration with high energy density at low temperatures.

**Figure 1.**
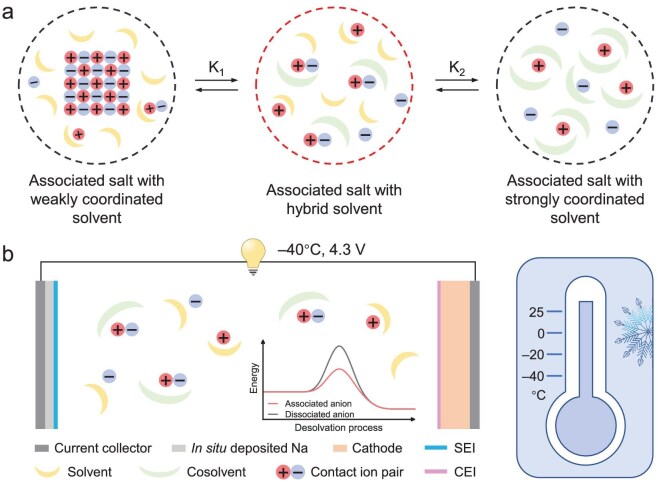
Electrolyte design principle of high-energy-density anode-free batteries at low temperatures. (a) The scheme of manipulating association-dissociation equilibrium. K is the chemical equilibrium constant. (b) Schematic illustration of the high-voltage anode-free Na battery working at low temperatures with the optimized electrolyte. Inset: the desolvation energy of the electrolyte containing associated anion compared with dissociated anion.

Herein, a high-energy-density and low-temperature anode-free Na battery is realized by manipulating the electrolyte association-dissociation equilibrium. Through selecting an associated salt (NaBF_4_) with high electrochemical stability and a hybrid solvent consisting of a low-freezing-point solvent (diglyme, G2) and a coordination-site-rich cosolvent (tetraglyme, G4), the salt association-dissociation equilibrium in the electrolyte is shifted from the associated state toward the CIP state, rather than the dissociated state (Fig. 1a). This generates more charge carriers, which increases the low-temperature ionic conductivity and forms more CIP-rich solvation structures, which lowers desolvation energy and facilitates the formation of inorganic-rich electrode/electrolyte interphases in both anode and cathode at low temperatures (Fig. 1b). Consequently, an ultrahigh CE of 99.90% in Na||carbon coated Al foil (Al@C) half batteries and 4.3 V Al@C||NaNi_1/3_Fe_1/3_Mn_1/3_O_2_ (NFM) anode-free full cells were achieved at −40°C. More importantly, an Ah-level anode-free pouch cell has been successfully fabricated, delivering a record-high energy density of 204 Wh kg^−1^ (based on weight of the entire pouch cell) at −40°C among reported low-temperature rechargeable batteries.

## RESULTS AND DISCUSSION

### Electrolyte design for high-voltage low-temperature anode-free batteries

**Figure 2.**
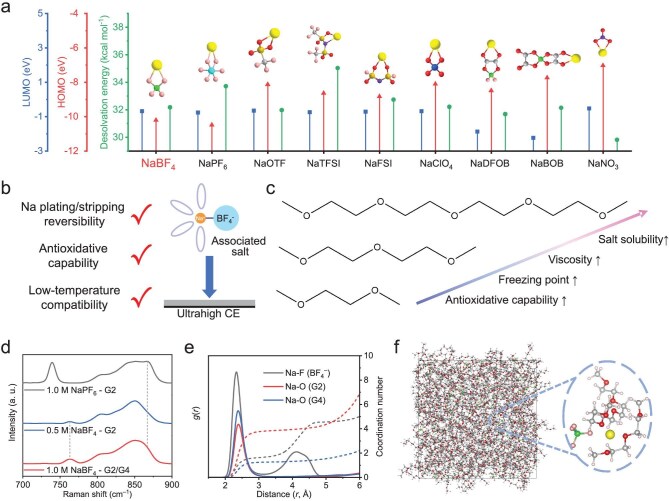
Electrolyte design for the high-voltage anode-free cells operating at low temperatures. (a) HOMO/LUMO energy level, and desolvation energy of various Na salts. Grey: C, red: O, purple: N, cyan: P, blue: Cl, pink: F, yellow: Na, green: B, khaki: S. (b) Advantages of NaBF_4_ salt. (c) Characteristics of glyme analogues. (d) Raman spectra of 1.0 M NaPF_6_-G2, 0.5 M NaBF_4_-G2, and 1.0 M NaBF_4_-G2/G4 electrolytes. (e) RDFs and coordination numbers of 1.0 M NaBF_4_-G2/G4 electrolyte collected from MD simulation, and (f) corresponding snapshot.

First, the favorable properties of the electrolyte salt for high-volage anode-free Na batteries at low temperatures are considered from the perspectives of both kinetic performance and thermodynamic stability. Since the desolvation process is generally considered as a rate-determining step in cryogenic batteries, the solvation structures, especially ion-dipole interactions between Na^+^ and solvents, largely determine their interfacial kinetics [22,23]:


(1) \begin{eqnarray*}
{U_{{\mathrm{ion}}-{\mathrm{dipole}}}} = - \frac{1}{{4\pi \varepsilon }}\frac{{ze\mu {\mathrm{\,\,cos}}\theta }}{{{r^2}}},
\end{eqnarray*}

where ${U_{{\mathrm{ion}}-{\mathrm{dipole}}}}$ is the ion-dipole force, $\varepsilon $ is the dielectric constant, $ze$ is the charge of the ion, $\mu $ is the dipole moment,$\,\,r$ is the distance between ions or the centers of dipoles, $\theta $ is the dipole angle relative to the line joining the ion and the center of the dipole. As illustrated in Equation (1), ${U_{{\mathrm{ion}}-{\mathrm{dipole}}}}$ can be reduced in conjunction with an increase in *r*. As the anions with a strong electron donor can lower the surface charge density of Na^+^ and lead to a larger bond length (*r*) between Na^+^ and solvent molecule [6], the highly associated Na salt is able to reduce ${U_{{\mathrm{N}}{{\mathrm{a}}^ + }-{\mathrm{solvent}}}}$ and is positively correlated with low solvation energy (Fig. 2a and Fig. S1). Therefore, highly associated Na salts are theoretically condusive to fast Na metal deposition kinetics in the anode side at low temperature. By screening numerous Na salts, the associated Na salts, including NaBF_4_, NaOTF, NaBOB, NaDFOB, and NaNO_3_ [24–26], exhibit higher donor numbers (Table S1) and low desolvation energies (Fig. 2b), while the dissociated salts, such as the most widely used NaPF_6_, exhibit the opposite. It is worth noting that the introduction of associated salt can also lower the freezing point of the Na-solvent–anion complex, which benefits ion migration in cold environments [25]. Furthermore, high thermodynamic stability of the electrolyte salts is necessary for their high-voltage application. Among highly associated salts, the NaBF_4_ and its solvation structure possess relatively low highest occupied molecular orbital (HOMO) and high lowest unoccupied molecular orbital (LUMO) energy level, suggesting its high theoretical oxidation and reduction resistance (Figs S2 and S3). In this view, NaBF_4_ is a promising candidate as the electrolyte salt for high-voltage anode-free batteries operating at low temperatures.

The solvents for the electrolyte should be selected to properly coordinate with the associated salt so as to achieve satisfactory electrolyte performance. To ensure the reversibility of Na plating/stripping at low temperatures, G2 was selected as the main solvent owing to its low freezing point (−64°C) and good compatibility with Na metal [27]. Nevertheless, the strong association between Na^+^ and BF_4_^−^ restricts the solubility of NaBF_4_ in G2 solvent (<0.64 M) [28], which limits the ionic conductivity of NaBF_4_-based electrolyte. Fortunately, the solubility can be improved by manipulating the dissociation–association equilibrium (Equation (2)) [29,30], and the ion pairs in an arbitrarily chosen solvent can be considered as:


(2) \begin{eqnarray*}
{\mathrm{N}}{{\mathrm{a}}^ + }{\mathrm{BF}}_4^ - \mathop \leftrightarrow \limits^{{\mathrm{sol}}} {\mathrm{\,\,}}{({\mathrm{N}}{{\mathrm{a}}^ + }{\mathrm{BF}}_4^ - )_{{\mathrm{sol}}}}\mathop \leftrightarrow \limits^{{\mathrm{sol}}} {\mathrm{\,\,}}{({\mathrm{N}}{{\mathrm{a}}^ + })_{{\mathrm{sol}}}} + {\mathrm{BF}}_4^ - ,
\end{eqnarray*}

where the associated solid salt is denoted by ${\mathrm{N}}{{\mathrm{a}}^ + }{\mathrm{BF}}_4^ - $, CIP by ${({\mathrm{N}}{{\mathrm{a}}^ + }{\mathrm{BF}}_4^ - )_{{\mathrm{sol}}}}$, and the fully dissociated ion pair by ${({\mathrm{N}}{{\mathrm{a}}^ + })_{{\mathrm{sol}}}} + {\mathrm{BF}}_4^ - $. The ideal electrolyte formulation for low-temperature operation is expected to increase the concentration of charge carriers with maintained CIP solvation structure [21]. In this consideration, G4 with rich coordination sites as well as similar dielectric constant and donor number to G2 (Table S2), was introduced in NaBF_4_-G2 electrolyte as the equilibrium-regulation cosolvent [31–33]. It would theoretically increase the solubility of NaBF_4_ without changing the dissociation state of ${({\mathrm{N}}{{\mathrm{a}}^ + }{\mathrm{BF}}_4^ - )_{{\mathrm{sol}}}}$ (Fig. 2c and Fig. S4). The above analysis was verified by experiments that showed the 1.0 M NaBF_4_-G2/G4 electrolyte achieves a high conductivity of 1.35 mS cm^−1^ at −40°C, which is higher than that of the typical dissociated-salt-based electrolyte frozen at −40°C (1.0 M NaPF_6_-G2) and the 0.5 M NaBF_4_-G2 electrolyte with limited concentration (Figs S5 and S6). Of note, the 1.0 M NaBF_4_-G2/G4 exhibits a high electrochemical stability up to 4.6 V (Fig. S7).

The solvation structure of the dissociation–association equilibrium-shifted electrolyte was further analyzed by Raman spectra. The peak at ∼760 cm^−1^, denoted as the Na^+^-anion coordination [13], exhibits a lower redshift in BF_4_^−^-based electrolyte than that of PF_6_^−^, indicating a strong interaction between Na^+^ and BF_4_^−^, which suggests higher CIP content. It is worth noting that, after the introduction of 20 vol% G4 cosolvent in NaBF_4_-G2 electrolyte, the position of this peak did not change, and such a strong interaction between cations and anions can be well preserved (Fig. 2d). In addition, the weaker Na^+^-glyme(O) coordination peak at 870 cm^−1^ in NaBF_4_-based electrolytes rather than the NaPF_6_ counterpart also suggests a weaker solvation effect between Na^+^ and solvents. Moreover, the solvation structure of these electrolytes was further analyzed by nuclear magnetic resonance (NMR) spectra (Fig. S8). Compared with NaPF_6_-based electrolyte, the Na peaks in the ^23^Na spectra in NaBF_4_-based electrolytes exhibit an upfield shift, indicating an enhanced Na^+^-anion interaction [14]. Further, molecular dynamic (MD) simulations were carried out to clarify the solvation structure in molecular sight. This can be seen in radial distribution function (RDF) plots (Fig. 2e, f, Figs S9 and S10). The solvents in the first solvation shell of Na^+^ (Na-O) delivers a lower coordination number of 5.02 in 1.0 M NaBF_4_-G2/G4 electrolyte than 1.0 M NaPF_6_-G2 and 0.5 M NaBF_4_-G2 electrolytes. Therefore, the electrolyte dissociation–association equilibrium is successfully manipulated by combining associated salt and hybrid solvent with appropriate coordination ability. It combines the weak solvation effect with high redox stability, which is expected to achieve low-temperature and high-voltage operation of anode-free batteries.

### Electrochemical characterization of Na plating and stripping

**Figure 3.**
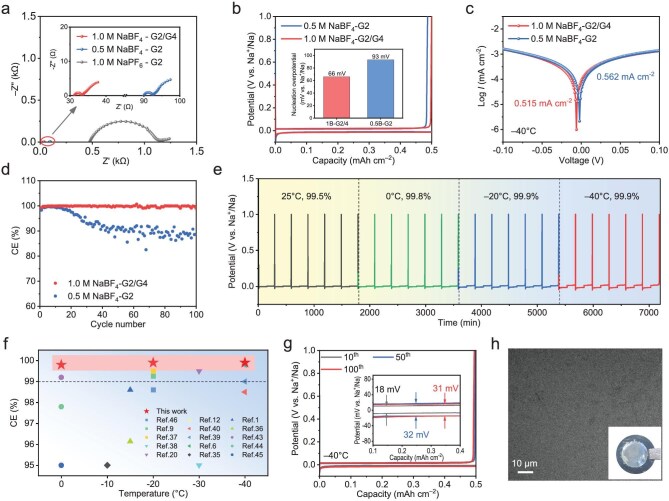
Low-temperature electrochemical performance of Na metal in different electrolytes. (a) Nyquist plots of Na||Na symmetry batteries in the electrolytes of 1.0 M NaPF_6_-G2, 0.5 M NaBF_4_-G2, and 1.0 M NaBF_4_-G2/G4 at −40°C. (b) Charge/discharge curves of Na||Al@C half cells with plating capacity of 0.5 mAh cm^−2^ and current density of 0.2 mA cm^−2^ at −40°C. Inset: nucleation overpotentials. (c) Tafel plots of Na||Na symmetry batteries in the electrolytes of 0.5 M NaBF_4_-G2 and 1.0 M NaBF_4_-G2/G4 at −40°C. (d) CEs of Na||Al@C half cells based on 0.5 M NaBF_4_-G2, and 1.0 M NaBF_4_-G2/G4 electrolyte at −40°C during cycling with 0.5 mAh cm^−2^ and 0.2 mA cm^−2^. (e) CEs of Na||Al@C half cells based on 1.0 M NaBF_4_-G2/G4 electrolyte at 25, 0, −20, and −40°C with 0.5 mAh cm^−2^ and 0.2 mA cm^−2^. (f) The CEs of Na||Al@C half cells comparison among the state-of-art alkali metal-based half cells at −40°C. (g) Voltage profiles of Na||Al@C half cells with different cycles in 1.0 M NaBF_4_-G2/G4 electrolytes at −40°C. (h) SEM images of the deposited Na dissembled from Na||Al@C half cells after 5 cycles at −40°C with 0.5 mAh cm^−2^ and 0.2 mA cm^−2^. Inset: digital photograph of deposited Na on Al@C.

Next, the performance of the NaBF_4_-based CIP-rich electrolyte for low-temperature Na metal plating/stripping reversibility is evaluated in Na–metal-based symmetric and half cells. Electrochemical impedance spectroscopy (EIS) measurement of the Na||Na symmetric cells with 1.0 M NaBF_4_-G2/G4 electrolyte exhibits an extremely low charge transfer resistance (1.98 ${\mathrm{\Omega }}$) at −40°C (Fig. 3a). Such fast charge transfer kinetics contribute to a low Na nucleation overpotential in Na||Al@C half cells (Fig. 3b, Figs S11 and S12). Moreover, the Tafel plots of the Na metal anode in 1.0 M NaBF_4_-G2/G4 electrolyte at −40°C exhibits a lower corrosion current compared with that in the G4-free electrolyte, which can be related to the reduced side effects of the designed electrolyte (Fig. 3c) [34]. As a result, an ultrahigh CE of 99.90% can be achieved by utilizing NaBF_4_-G2/G4 electrolyte at −40°C, and it maintains stability in a wide temperature range, long-time cycling, and large plating/stripping capacity (Fig. 3d, e and Fig. S13). Such high CEs set a record in low-temperature alkali metal batteries (Fig. 3f) [1,6,8,9,12,20,35–46]. Moreover, the overpotential only increased by 7 mV within 100 cycles and keeps in a low value of 16 mV at the 100th cycle (Fig. 3g), suggesting negligible accumulation of by-products. Additionally, a uniform and smooth morphology can be observed in scanning electron microscopy (SEM) image of the deposited Na at −40°C (Fig. 3h). This high reversibility of Na plating/stripping in the CIP-rich NaBF_4_-G2/G4 electrolyte supports the realization of anode-free Na batteries at low temperatures.

### Experimental and theoretical analysis of SEI and CEI

**Figure 4.**
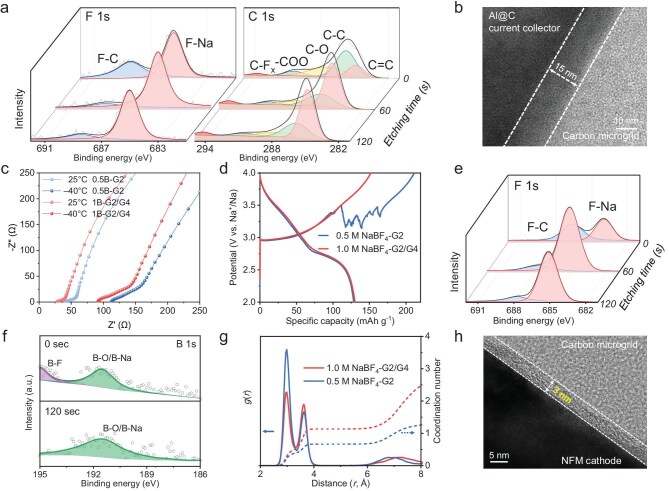
Experimental and theoretical analysis of SEI and CEI. (a) F 1 s and C 1 s XPS spectra of the SEI formed on cycled Al@C at − 40°C recorded at different sputtering times, and (b) the corresponding TEM image. (c) Nyquist plots of Na||NFM half cells after cycling at a discharged state. (d) Voltage profiles of Na||NFM half cells in different electrolytes. (e) F 1 s and (f) B 1 s XPS spectra at different sputtering times of CEI layers formed on NFM cathode after cycling at −40°C. (g) RDFs of 0.5 M NaBF_4_-G2 and 1.0 M NaBF_4_-G2/G4 at −40°C. (h) TEM image of the CEI layer formed on NFM cathode.

To elucidate the mechanism for the enhanced reversibility of Na plating/stripping, the chemical composition of SEI on the Al@C surface was investigated using X-ray photoelectron spectroscopy (XPS) with different sputtering times (Fig. 4a) using a semi *in situ* vacuum transfer device (Fig. S14). In F 1s and C 1s spectra, the signals of F–Na and C–F_x_ were enhanced with the etching depth, indicating that a NaF-rich phase exists in the inner SEI layer. Such an inorganic NaF component generally shows a high mechanical strength and can withstand the volume change of Na plating and stripping [1,9]. Additionally, the transmission electron microscopy (TEM) image of their surface morphology exhibits a uniform SEI with a thickness of ∼15 nm on Al@C (Fig. 4b and Fig. S15).

Apart from reversibility of the anode, the stable operation of energy-dense anode-free full cells greatly depends on the high-voltage stability of the cathode interphase [13]. The Na||NFM half cells based on 1.0 M NaBF_4_-G2/G4 electrolyte exhibit low charge transfer resistances of cathodes during low-temperature cycles without continuous electrolyte decomposition at high voltage (Fig. 4c and d) [3]. Further, the XPS spectra collected from CEI formed on NFM cathodes (Fig. 4e and f) show the existence of inorganic components, including F–Na, B–F, and B–O species. Among them, B–O species with poor crystallinity, which act as the glass components, are able to improve the flexibility and robustness of CEI [5]. To further investigate the mechanism of CEI formation, MD stimulations were performed (Fig. 4g). The RDFs of Na–F reveal a larger coordination number in 1.0 M NaBF_4_-G2/G4 electrolyte than that in 0.5 M NaBF_4_-G2 electrolyte, which favors a preferential decomposition of NaBF_4_ to construct the inorganic-rich sustainable CEI. Moreover, the high-resolution TEM image of the cathode displays a uniform and ultrathin CEI film of ∼3 nm, leading to a low interfacial impedance at a low temperature (Fig. 4h). Therefore, the thin and robust passivation layers of SEI and CEI guarantee the stable operation of anode-free batteries under cryogenic conditions.

### Electrochemical performance of the anode-free full cells at low temperatures

**Figure 5.**
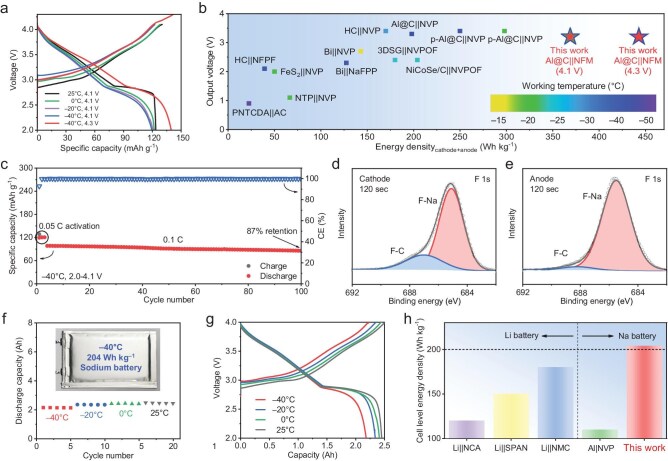
Electrochemical performance of the anode-free full cells at low temperatures. (a) Voltage profiles of anode-free Al@C||NFM full cells at different temperatures and voltage cut-off. (b) Energy density (based on the mass of active materials in cathode and anode) and output voltage comparison of low-temperature Na full cells. (c) Cycling performance of Al@C||NFM full cells at −40°C (1 C = 150 mA g^−1^, voltage cut-off: 2.0–4.1 V). F 1s XPS spectra of the (d) CEI and (e) SEI formed in the cycled anode-free full cells at −40°C and recorded at a sputtering time of 120 sec. (f) Discharge capacity of anode-free pouch cells at different temperatures. Inset: digital photograph of the pouch cell. (g) Voltage profiles of anode-free pouch cells with charge and discharge currents of 100 mA at 250°C and 50 mA at −20/−40°C (voltage cut-off: 2.0–4.0 V). (h) Energy density (based on the mass of entire pouch cell) comparison of state-of-the-art pouch cells at −40°C.

Based on the superior electrolyte that combines high Na metal compatibility and high-voltage stability under cold conditions, an energy-dense low-temperature anode-free battery was fabricated. Given the high sensitivity of anode-free full cells to water (Fig. S16), 2 vol% polydimethylsiloxane (PDMS) was introduced into the electrolyte to further remove the trace water and hydrofluoric acid (HF) [47]. It should be noted that the low concentration of PDMS in full cells does not affect the solvation structure or stability in both anode and cathode (Figs S17–S20). The anode-free Al@C||NFM full cells exhibit high capacities of 123.8 mAh g^−1^, 121.9 mAh g^−1^, 120.1 mAh g^−1^, 119.2 mAh g^−1^ at 25, 0, −20, and −40°C, respectively, with a voltage cut-off of 4.1 V (Fig. 5a and Figs S21–S28). Interestingly, when the voltage range broadens to 2.0–4.3 V, the anode-free battery exhibits a high capacity of 139.7 mAh g^−1^ at −40°C, and the energy density is up to 443 Wh kg^−1^ (based on the mass of active materials in cathode and anode), reaching a record-high value in reported low-temperature Na full cells (Fig. 5b) [6,12,48–55]. After 100 cycles, the capacity maintains 87% of the initial cycles at −40°C (Fig. 5c and Fig. S29), which can be attributed to the well-maintained inorganic-rich electrode-electrolyte interphase in anode-free full cells during cycling (Fig. 5d, e, Figs S30 and S31). To reveal the capacity degradation mechanisms, the anode-free full cell was disassembled after long cycling at a discharged state. The irreversible Na metal and transition metal species (Ni, Fe, Mn) dissolution and shuttle from the NFM cathode have been observed on the Al@C current collector (Fig. S32), which jointly cause the capacity decay during cycling. Furthermore, benefitting from the facile manufacture of anode-free batteries, the anode-free pouch cells were assembled (Fig. 5f, g, Fig. S33, and Table S3). Importantly, the Ah-level pouch cells reached high energy densities of 236, 231, 225 and 204 Wh kg^−1^ (based on the mass of the entire cell) at 25, 0, −20, and −40°C, respectively, exceeding previously reported Na pouch cells. More impressively, the energy density of the as-prepared anode-free pouch cells at −40°C even surpasses state-of-the-art Li-based batteries (Fig. 5h) [6,22,39,56].

## CONCLUSIONS

In summary, a high-energy-density and low-temperature anode-free sodium battery was realized by manipulating association-dissociation equilibrium through rational electrolyte design. By selecting a highly associated salt (NaBF_4_) and a hybrid solvent (G2/G4) with appropriate coordination ability, the electrolyte association-dissociation equilibrium is shifted from the associated salt state to CIP-rich state, rather than the dissociated state. The CIP-rich solvation structure contributes to a reduced desolvation energy, higher ionic conductivity, and the formation of inorganic-rich SEI and CEI under cryogenic conditions. Thus, the low-temperature kinetics and high-voltage stability of the battery were simultaneously promoted. Owing to these synergies, the 4.3 V Al@C||NFM full cells achieve a high energy density of 443 Wh kg^−1^_cathode+anode_ at −40°C. Impressively, the Ah-level anode-free pouch cells achieved high energy densities of 236, 231, 225 and 204 Wh kg^−1^ (based on the mass of the entire cell) at 25, 0, −20, and −40°C, respectively, surpassing reported Na pouch cells. Specifically, the energy density of the pouch cells at −40°C even exceeds state-of-the-art low-temperature Li batteries. This work presents a significant advancement in the design of high-energy-density low-temperature batteries, both in concept and demonstration.

## Supplementary Material

nwaf124_Supplemental_File
