# Supplementary material for: A >200 Wh kg−1 anode-free Na pouch battery at −40°C enabled by manipulating electrolyte equilibrium
Source: Natl Sci Rev. 2025 Mar 29;12(6):nwaf124. doi: 10.1093/nsr/nwaf124 (PMC12060854; doi:10.1093/nsr/nwaf124)
Supplement: nwaf124_Supplemental_File [file nwaf124_supplemental_file.pdf]

Supplementary Data

A >200 Wh kg^{-1} anode-free Na pouch battery at −40 ℃ enabled by manipulating electrolyte equilibrium

Qiaonan Zhu[1], Jiawei Wang[2], Liqiang Wu[3], Hao Lan[1], Jiangchun Chen[1], Liwei Cheng[1], Bin Zhou[3], Daojun Yang[3], Jie Yang[4], Mirtemir Kurbanov[5], Shuai Dong[1,6*], Hua Wang[1*]

[1]School of Chemistry, Beihang University, Beijing 100191, China

[2]School of Materials and Chemistry, University of Shanghai for Science and Technology, Shanghai 200093, China

[3]Beijing Xibei Power Technology Co., Ltd., Beijing 102600, China

[4]Hydrogen Energy Research Center, PetroChina Petrochemical Research Institute, Beijing 100083, China

[5]Arifov Institute of Ion-Plasma and Laser Technologies, Academy of Sciences of the Republic of Uzbekistan, Tashkent 100077, Uzbekistan

[6]School of Chemistry and Chemical Engineering, Henan Key Laboratory of Biomolecular Recognition and Sensing, Henan D&A Engineering Center of Advanced Battery Materials, Shangqiu Normal University, Shangqiu 476000, China

*Corresponding author: Hua Wang (wanghua8651@buaa.edu.cn); Shuai Dong (tiankong890528@126.com)

METHODS

Preparation of electrolyte

The electrolytes were prepared by dissolving n M (mol L^{-1}) $NaBF_4$ (Alfa, 97%) or $NaPF_6$ (Dodochem, 99%) in G2 (Aladdin, 99.5%) or G4 (Aladdin, 99%) solvents. The optimized electrolyte contains 1.0 M $NaBF_4$ dissolved in G2/G4 cosolvent (vol ratio: 8/2). It is worth noting that the G2/G4 cosolvent with volume ratio of 9/1 cannot dissolve 1.0 M $NaBF_4$ while that of 7/3 significantly increases the low-temperature overpotentials of Na plating/stripping (Figure S9). Therefore, G2/G4 (vol ratio: 8/2) was chosen as the optimized solvent. For anode-free full cells, 2 vol% PDMS (Alfa, M. W. 770) was dissolved in 1.0 M $NaBF_4$-G2/G4 electrolyte to remove the trace amounts of water and HF. All the electrolytes were dried using 4Å molecular sieves for 48h and the sodium metal foil (Aladdin, 99.7%) for another 48h in an Ar-filled glove box.

Electrochemical measurement

The cathode slurry composed of 80wt% NFM (GEM Co., Ltd.), 10wt% Super P (MTI Co., Ltd.) and 10wt% polyvinylidene fluoride (PVDF, MTI Co., Ltd.) was casted on the Al@C foil (Saibo electrochemistry, carbon loading: 0.05 mg cm^{-2}), followed by a drying process at 120 ℃ under vacuum (total mass loading: 6-8 mg cm^{-2}). The anode-free Al@C||NFM coin cells (CR2032) were assembled by pairing the NFM cathode (14 mm in diameter) with Al@C current collector (19 mm diameter) in an Ar-filled glovebox. Two layers of Celgard 2500 and one layer glass fiber membrane membranes were used as the separator, and the amount of electrolyte was 80 μL. The anode-free pouch cells were assembled in a dry room by using 11 layers of 4.5×9.0 cm^2 NFM cathode (active mass loading: 21 mg cm^{-2}) and 12 layers of 4.8×9.4 cm^2 Al@C current collector with 10 g (4 g Ah^{-1}) of the regulated electrolyte and 9 μm PE + 3μm boehmite membranes. For the Na||Al@C half cells and Na||Na symmetric cells, each cell was consisted of the Al@C (19 mm in diameter) and a Na foil counter electrode (~1.0 cm^2) with two layers of Celgard 2500 as the separator and 50 μL of electrolytes. The low-temperature electrochemical performance was tested on CT 3001A Land battery testing systems (LAND Electronic) in a cryogenic box (JK-80G, Kingjo). The anode-free cells were first cycled at 0.05 mA cm^{-2} from 1 to 2 V for 10 cycles to remove surface contamination, followed by cycling at various current densities. EIS was obtained in the frequency range of 10^6 Hz-10^{-2} Hz with an amplitude of 10

mV (1400 cell test system, Solartron). The ion conductivity (σ) can be calculated by the following equation:

$$\sigma = \frac{L}{SR} \#(1)$$

where L, S, and R represent the distance between two platinum (Pt) electrodes, the area of Pt electrodes, and the ionic resistance obtained from EIS measurement, respectively. LSV tests were performed with Na||Al@C coin cells on a CHI660E electrochemical station at a scan rate of 1 mV s^{-1}. Tafel plots were obtained in Na||Na coin cells on a CHI660E electrochemical station at a scan rate of 1 mV s^{-1}. The energy densities (E) were calculated by the following equation:

$$E_{cathode+anode} = \frac{I\frac{\int U(t)}{dt}}{m_{NFM}} \#(2)$$

$$E_{entire\ cell} = \frac{I\ \int U(t)\ /dt}{m_{entire\ cell}} \#(3)$$

where I is the average cell operating current; U is the operating voltage of the cell; m_{NFM} is the mass of active materials in cathode; The weight of active material in anode is 0; $m_{entire\ cell}$ is the total mass of the entire pouch cell.

Characterization

The solvation structures of the electrolytes were investigated by Raman spectra spectrometer (LabRAM HR) with 785 nm lasers. The ^{23}Na Nuclear magnetic resonance (NMR) spectra of electrolytes were collected on Bruker Ascend Evo 400 MHz. A capillary insert with 0.1 M $NaPF_6$ in the deuterated dimethyl sulfoxide (DMSO-d6) was applied as the reference. The morphologies of Na were observed by field-emission scanning electron microscopy (JEOL-7500), and the samples were sealed in an argon-filled box and then quickly loaded on the SEM holder. X-ray photoelectron spectroscopy (XPS) was performed by an ESCALAB 250Xi to characterize the compositions of SEI films. Sputter etching was conducted using an Ar^+ beam (10 kV, 100 nA) to obtain the depth profile. A semi-in-situ vacuum transfer device (XPS Cell-Xi, Beijing Billison Technology Co., Ltd.) was used before collecting XPS spectra. High-resolution transmission electron microscopy (HRTEM) was carried out on JEM F200. The charged and discharged electrodes were first disassembled in an Ar-filled glove box, then rinsed with DME solvent, and subsequently dried in vacuum transition chamber.

Computational method

Density functional theory (DFT) calculations were performed in the Gaussian 09 D.01 program using the PBE1PBE/Def2-TZVP energy level, and the solvent effects are considered by the implicit solvation model based on density (SMD) (diethyl ether, eps=7.4, epsinf=1.987). The optimized molecular structures visualized using Multiwfn[1] 3.7 and Visual Molecular Dynamics 1.93 software.[2] The desolvation energy (E) was calculated with the equation below:

$$E = E_{G2} + E_{Na\ salt} - E_{G2-Na\ salt} \#(4)$$

where $E_{G2-Na\ salt}$, E_{G2}, and $E_{Na\ salt}$ are the total energies of G2-Na salt complex, G2, and Na salt, respectively.

The MD calculations were performed to analyze the solvation structure of electrolytes by the GROMACS 5.3 software[3] with a force field of OPLS-AA. The 1.0 M $NaBF_4$-G2/G4 electrolyte simulation box includes 50 Na^+, 50 PF_6^-, 281 G2, and 45 G4 species; 0.5 M $NaBF_4$-G2 includes 25 Na^+, 25 PF_6^-, and 352 G2 species; 1.0 M $NaPF_6$-G2 includes 50 Na^+, 50 PF_6^-, and 352 G2 species. The molecules were firstly filled randomly and arranged periodically in the XYZ direction into a 43 × 43 × 43 Å cubic boxes with PACKMOL software. The electrolyte systems were equilibrated for 10 ps followed by a 1000 ps simulation run (time step: 2 fs) in an isothermal-isobaric ensemble (NPT). The temperature was controlled to 233 K by a V-rescale thermostat. VMD was used to analyze the radial distribution function (RDF) from the simulation trajectory.

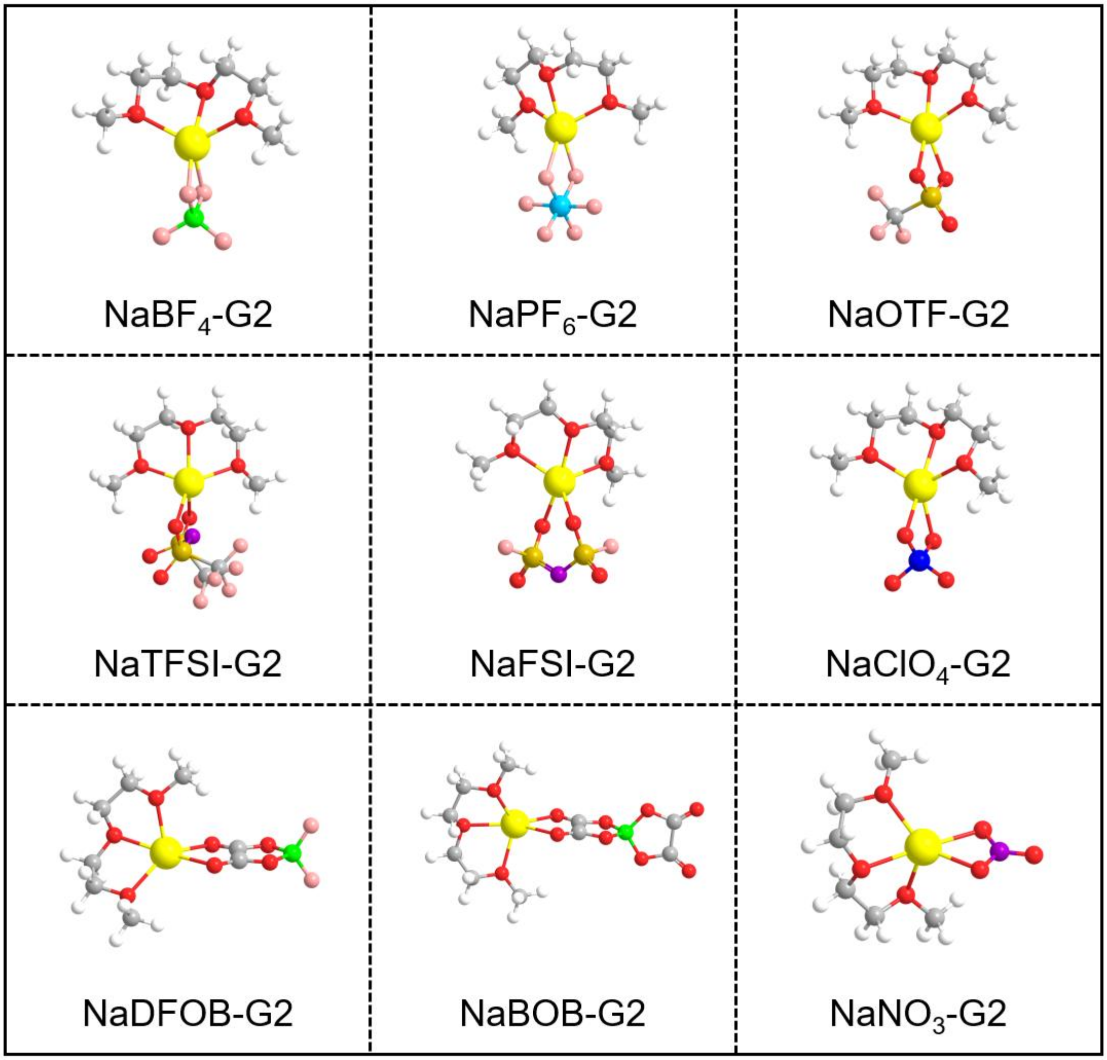

Figure S1. The optimized structure of salt-solvent complex. White: H, Grey: C, red: O, purple: N, cyan: P, blue: Cl, pink: F, yellow: Na, green: B, khaki: S.

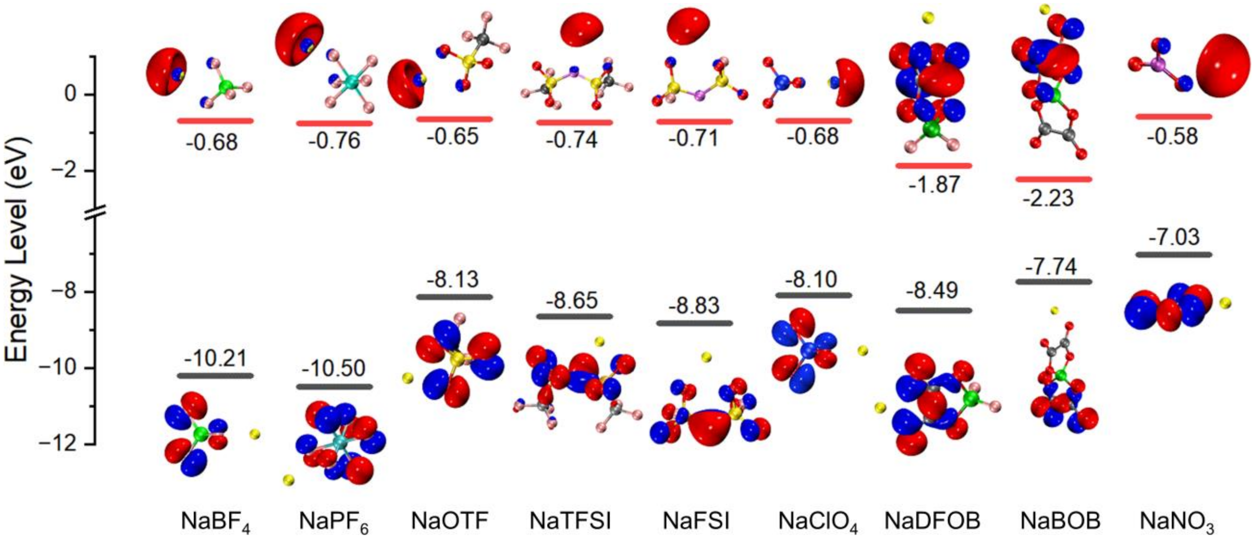

Figure S2. HOMO and LUMO structures and energy levels of various Na salts.

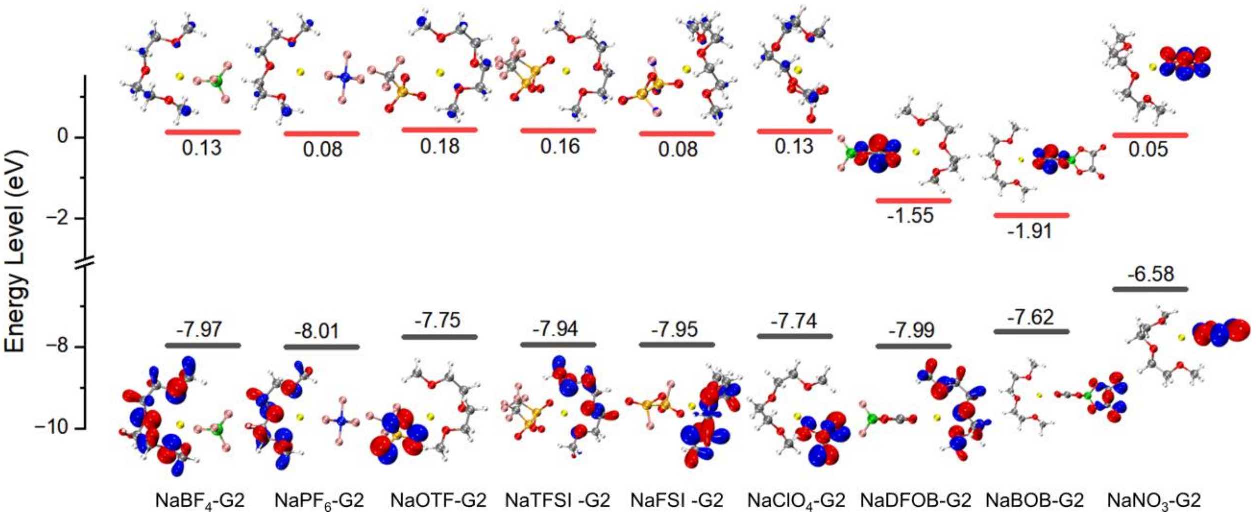

Figure S3. HOMO and LUMO structures and energy levels of various Na salts-G2 solvation complex.

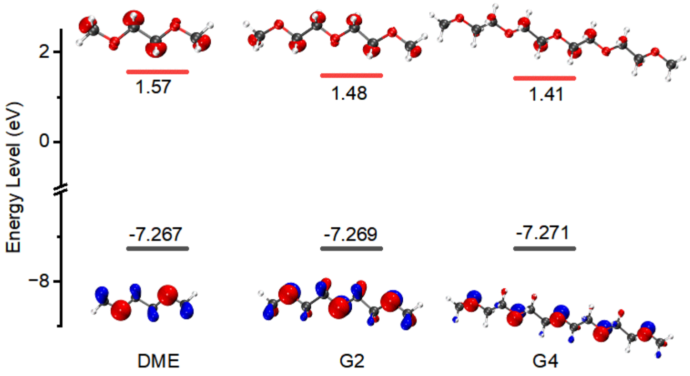

Figure S4. The optimized structure of solvents and corresponding LUMO/HOMO energy level.

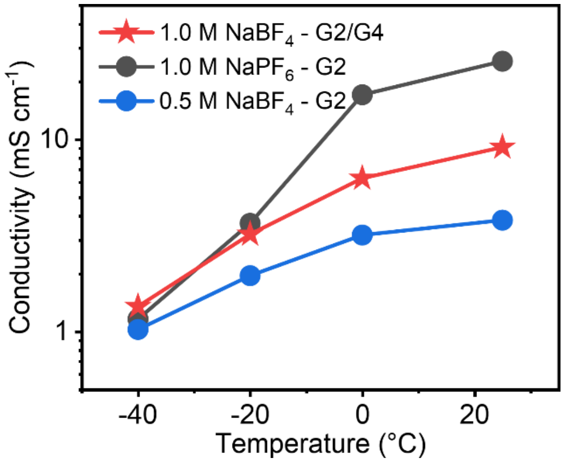

Figure S5. The dependence in ionic conductivities of different electrolytes on temperatures.

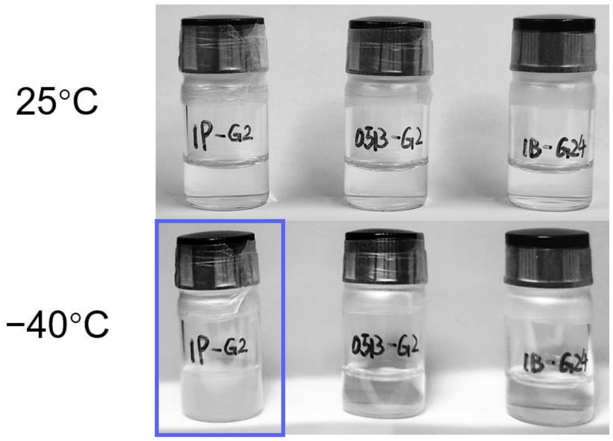

Figure S6. Photos of various electrolytes after resting at 25 ℃ (top) and −40 °C (bottom) for 12 h.

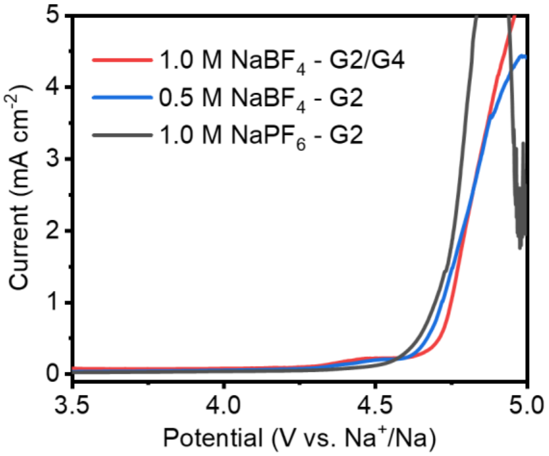

Figure S7. LSV curves obtained from the Na||Al@C half cells with different electrolytes at a scan rate of 1 mV s^{-1} under 25 ℃.

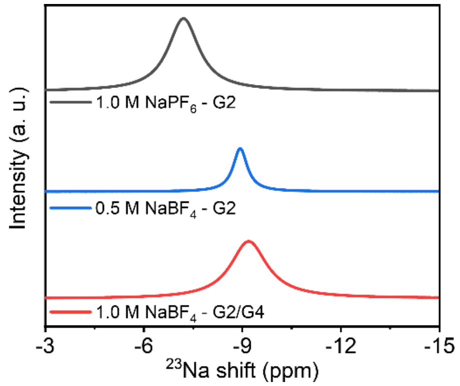

Figure S8. NMR ^{23}Na spectra of the 1.0 M $NaBF_4$-G2/G4, 0.5 M $NaBF_4$-G2, and 1.0 M $NaPF_6$-G2 electrolytes.

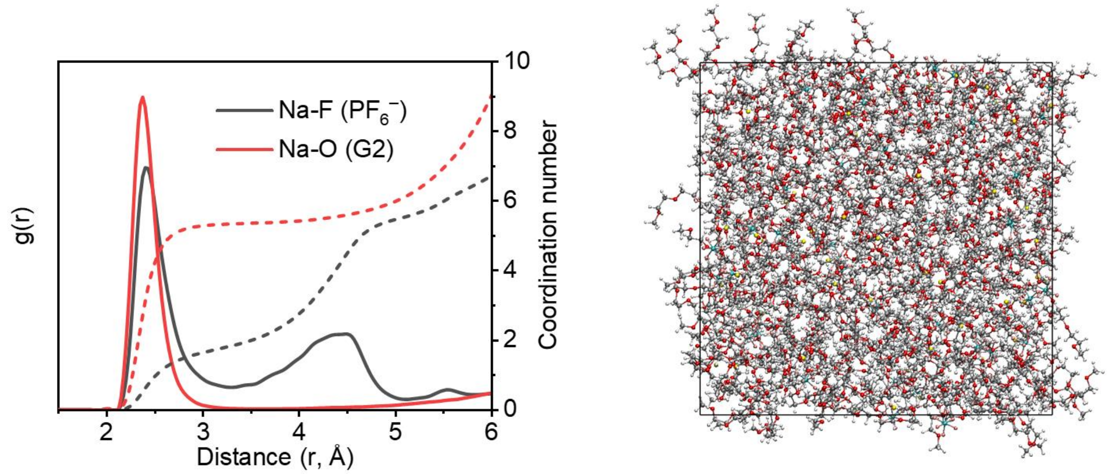

Figure S9. RDFs and coordination numbers of 1.0 M $NaPF_6$-G2 electrolyte collected from MD simulation and corresponding snapshot.

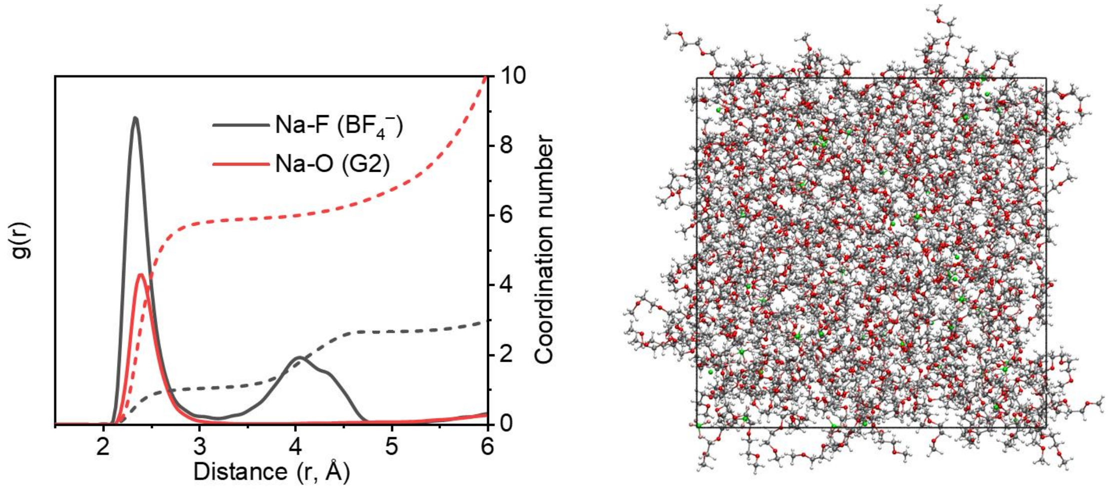

Figure S10. RDFs and coordination numbers of 0.5 M $NaBF_4$-G2 electrolyte collected from MD simulation and corresponding snapshot.

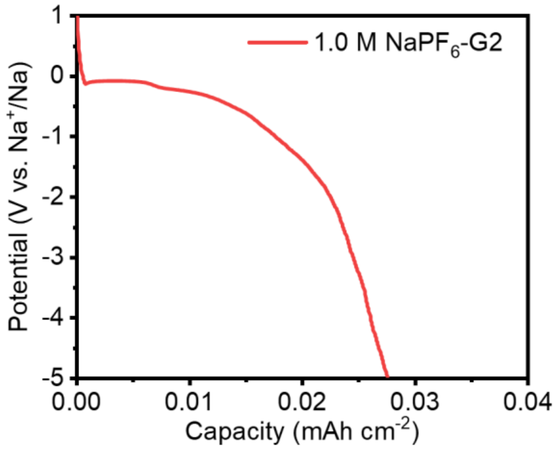

Figure S11. Voltage profiles of the Na||Al@C half cell based on the 1.0 M $NaPF_6$-G2 electrolyte at –40 ℃ with the current density of 0.2 mA cm^{-2}.

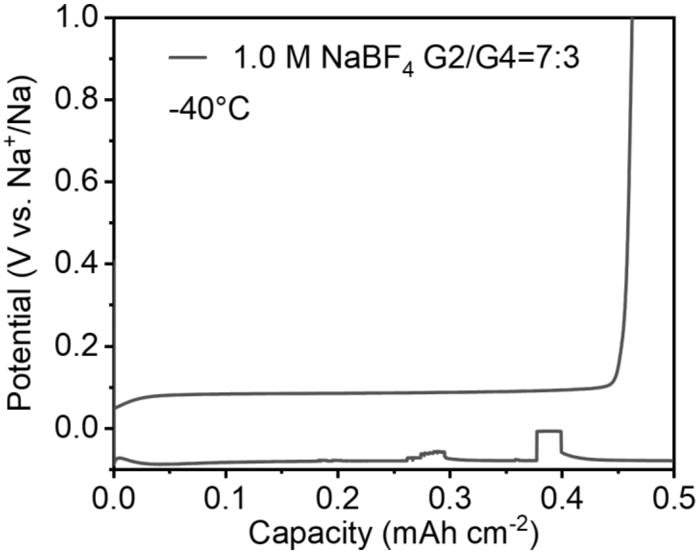

Figure S12. Voltage profiles of the Na||Al@C half cell based on the 1.0 M $NaBF_4$-G2/G4 (vol ratio: 7/3) electrolyte at –40 ℃ with the current density of 0.2 mA cm^{-2}.

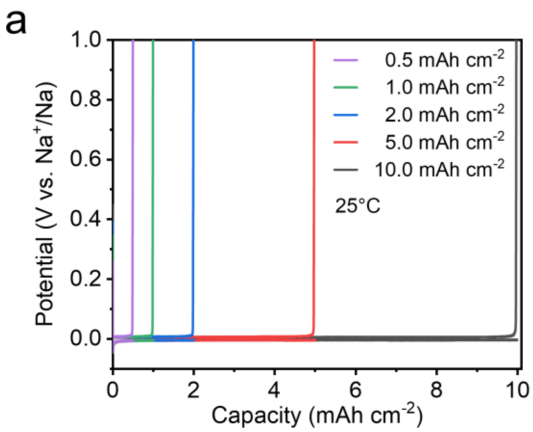

Figure S13. Voltage profiles of the Na||Al@C half cell based on the 1.0 M $NaBF_4$-G2/G4 electrolyte at 25 ℃ and –40 ℃ with the current density of 0.5 mA cm^{-2} and 0.2 mA cm^{-2}, respectively.

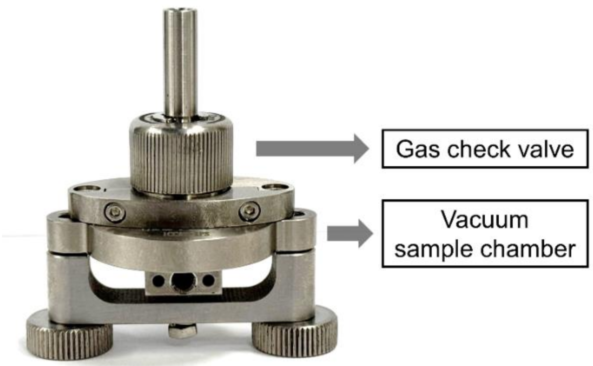

Figure S14. Digital photograph of *semi in-situ* vacuum transfer for XPS characterization.

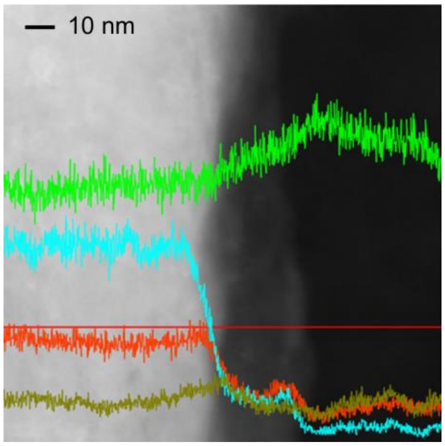

Figure S15. Line scan of the SEI film (green: C, red: O, blue: F, olive: Na).

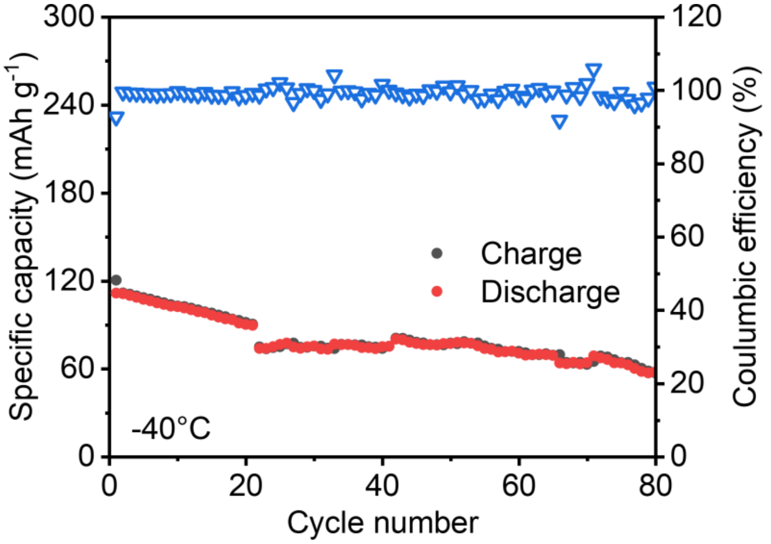

Figure S16. Cycling performance of Al@C||NFM full cells based on 1.0 M $NaBF_4$-G2/G4 at – 40 ℃.

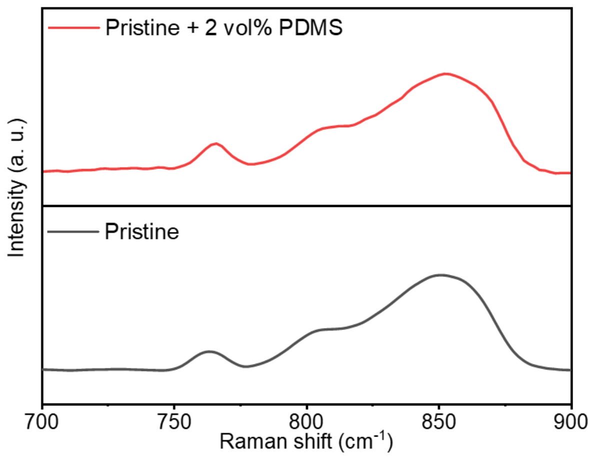

Figure S17. Raman spectra of 1.0 M $NaBF_4$-G2/G4 and 1.0 M $NaBF_4$-G2/G4+2 vol% PDMS electrolytes.

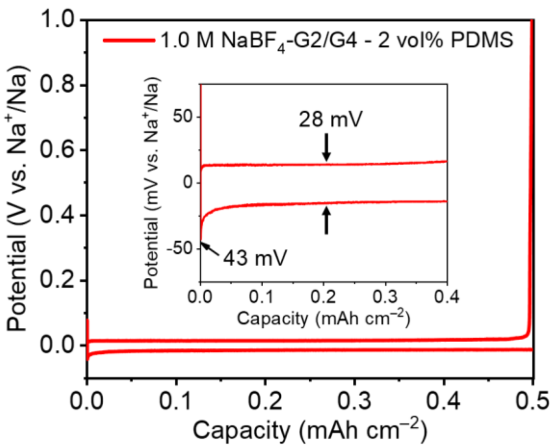

Figure S18. The charge/discharge curves of Na||Al@C half cells based on 1.0 M $NaBF_4$-G2/G4+2 vol% PDMS with the plating capacity of 0.5 mAh cm^{-2} and the current density of 0.2 mA cm^{-2} at –40 ℃.

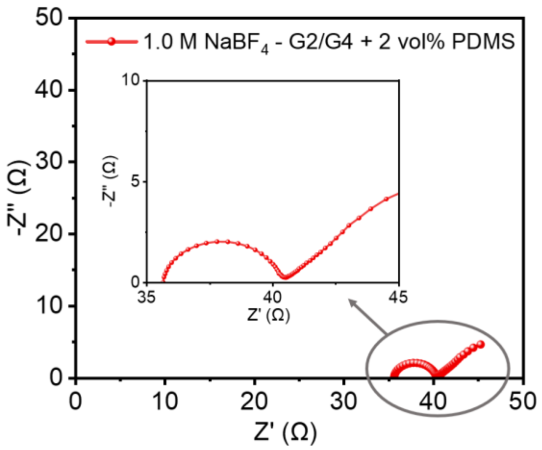

Figure S19. Nyquist plots of Na||Na symmetry batteries in the electrolytes of 1.0 M $NaBF_4$-G2/G4+2 vol% PDMS at –40 ℃.

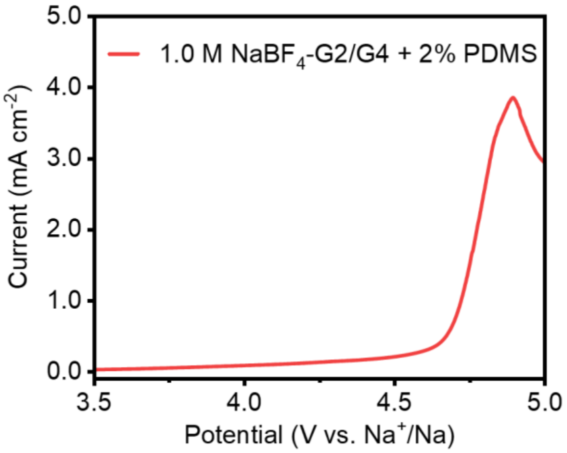

Figure S20. LSV curves obtained from the Na||Al@C half cell with 1.0 M $NaBF_4$-G2/G4+2 vol% PDMS electrolyte at a scan rate of 1 mV s^{-1} under 25 ℃.

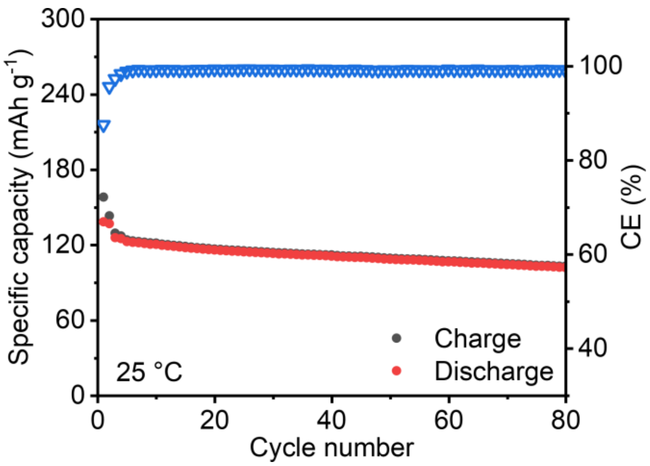

Figure S21. Cycling performance of Al@C||NFM full cells based on 1.0 M $NaBF_4$-G2/G4+2 vol% PDMS at 25 ℃.

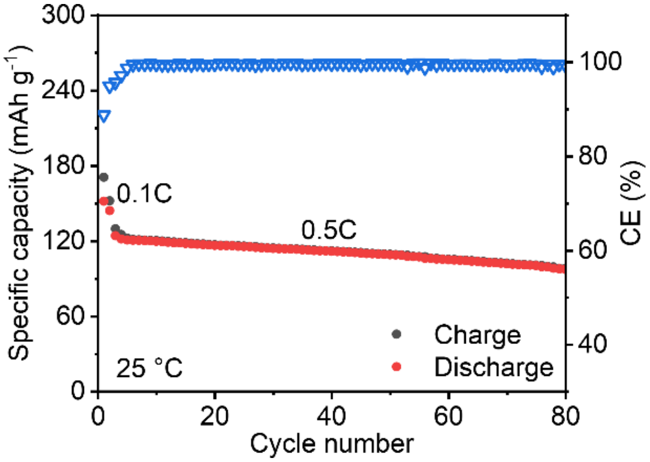

Figure S22. Cycling performance of Al@C||NFM full cells based on 1.0 M $NaPF_6$-G2 electrolyte at 25 ℃.

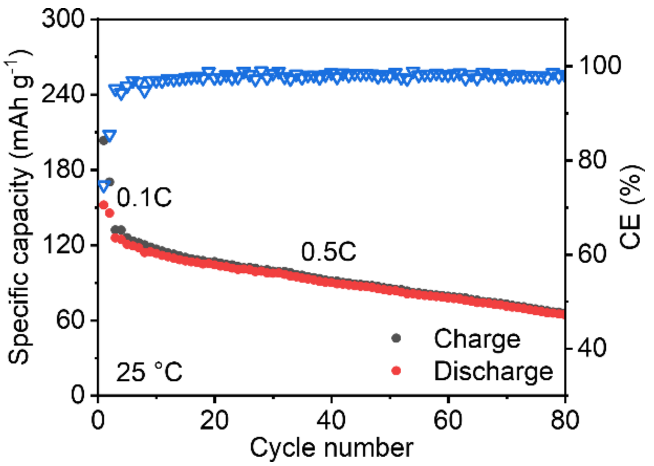

Figure S23. Cycling performance of Al@C||NFM full cells based on 0.5 M $NaBF_4$-G2 electrolyte at 25 ℃.

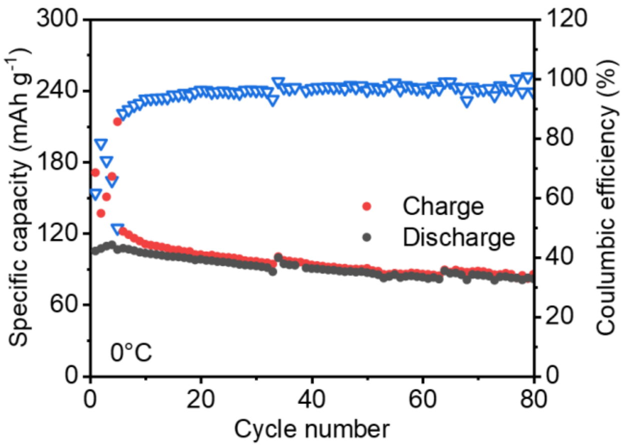

Figure S24. Cycling performance of Al@C||NFM full cells based on 1.0 M $NaBF_4$-G2/G4+2 vol% PDMS at 0 ℃.

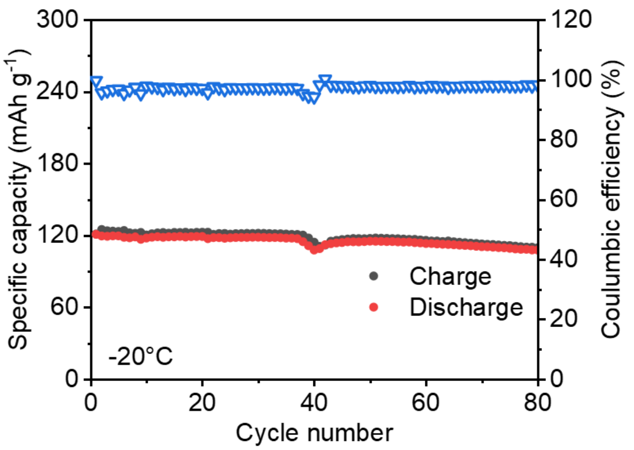

Figure S25. Cycling performance of Al@C||NFM full cells based on 1.0 M $NaBF_4$-G2/G4+2 vol% PDMS at −20 ℃.

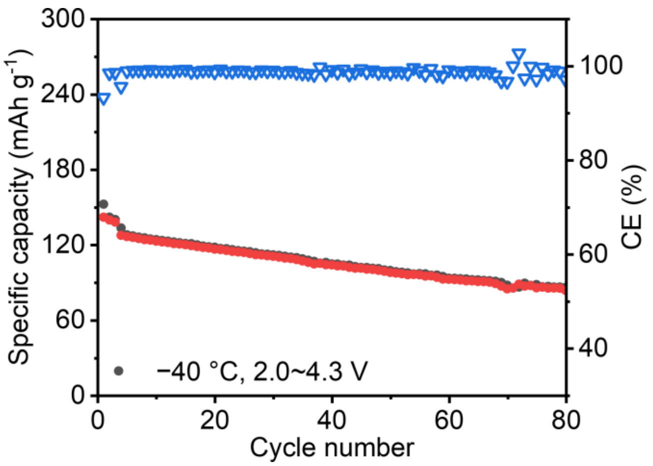

Figure S26. Cycling performance of Al@C||NFM full cells based on 1.0 M $NaBF_4$-G2/G4+2 vol% PDMS at −40 ℃ with a voltage cut-off of 2.0~4.3 V.

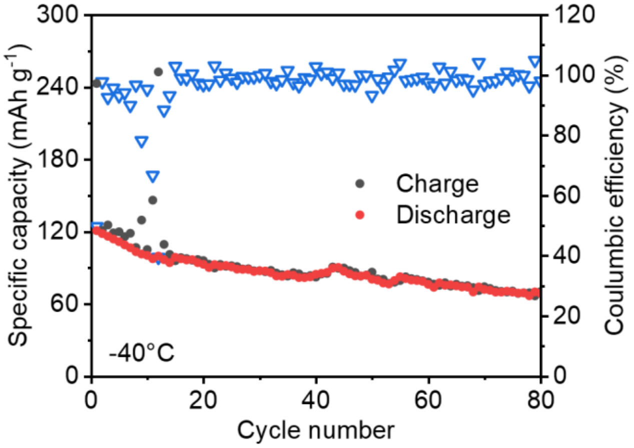

Figure S27. Cycling performance of Al@C||NFM full cells based on 0.5 M $NaBF_4$-G2 at −40 ℃.

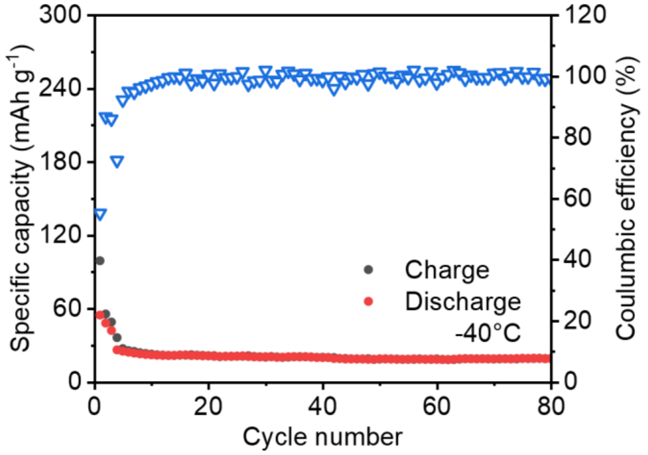

Figure S28. Cycling performance of Al@C||NFM full cells based on 1.0 M $NaPF_6$-G2 at −40 ℃.

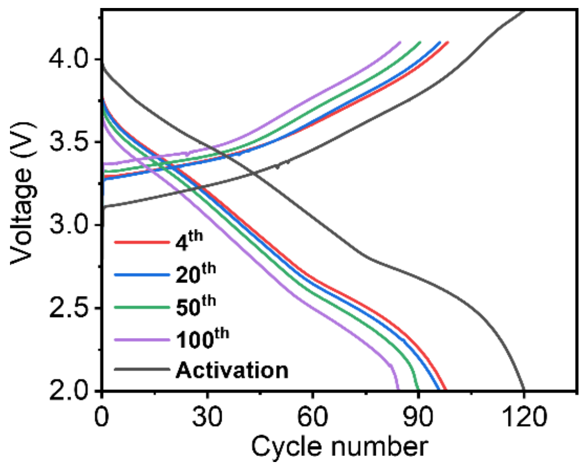

Figure S29. The charge/discharge curves of the p-Al@C||NVP cell based on 1.0 M $NaBF_4$ G2/G4 + 2%PDMS at –40 ℃.

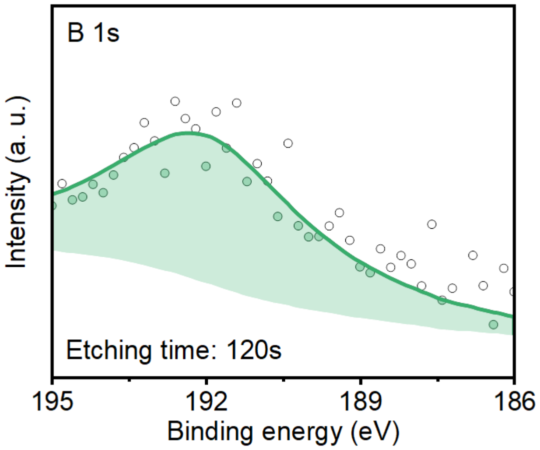

Figure S30. B 1s XPS spectra of CEI layers formed on NFM in Al@C||NFM full cell at −40 ℃.

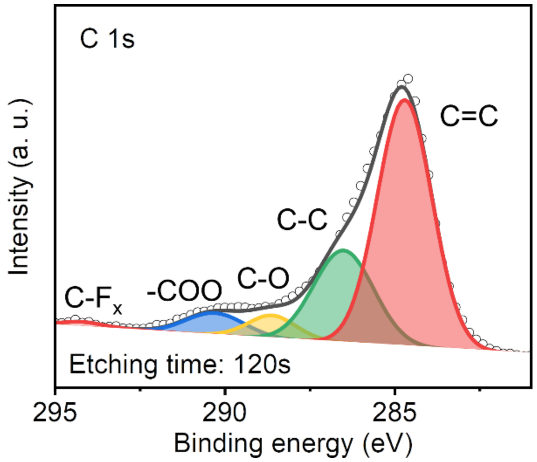

Figure S31. C 1s XPS spectra of SEI layers formed on Al@C in Al@C||NFM full cell at −40 ℃.

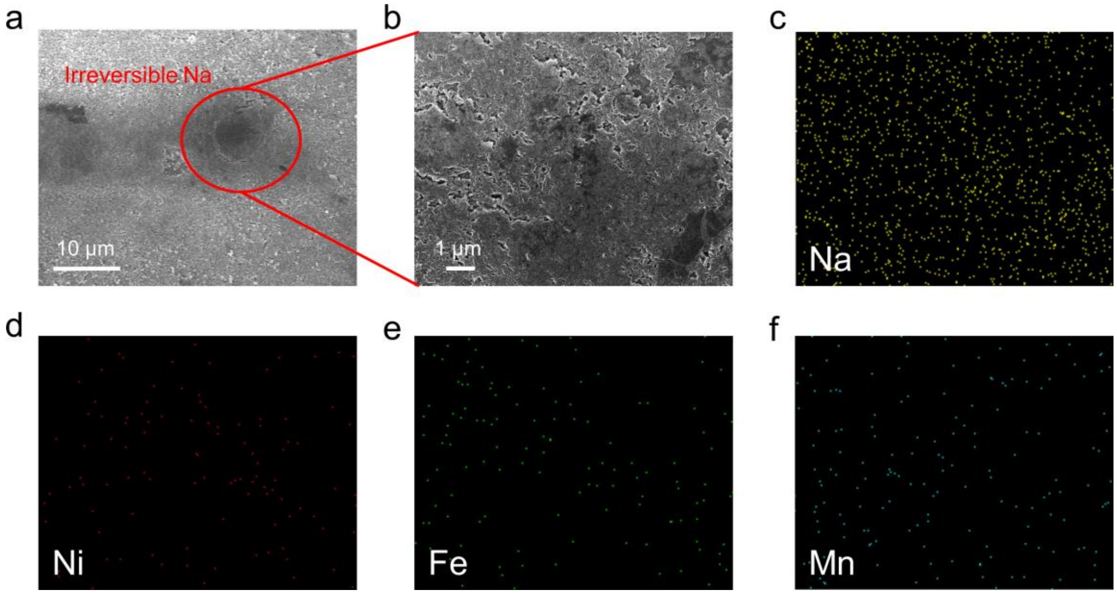

Figure S32. (a, b) The SEM images of Al@C in Al@C||NFM full cell after cycles, and corresponding EDS mapping images of (c) Na, (d) Ni, (e) Fe, (f) Mn.

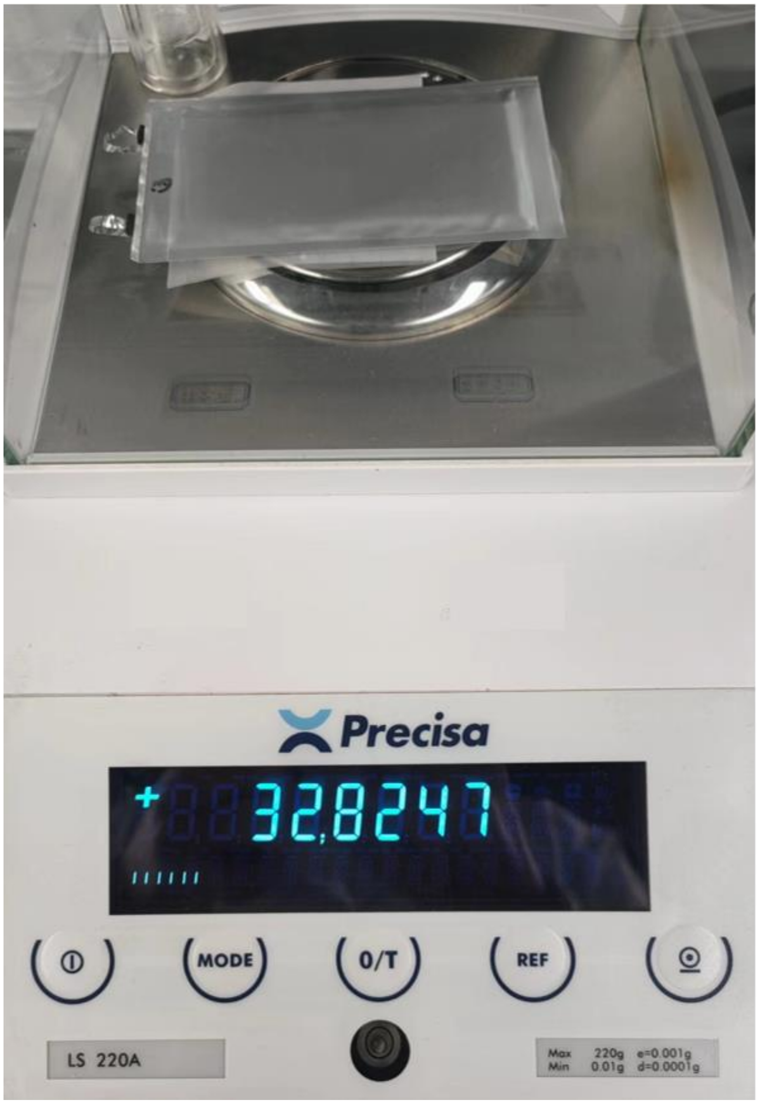

Figure S33. Digital photography of the anode-free Al@C||NFM pouch cell. The weight of the entire cell is 32.8247 g.

Table S1. Donor number of various anions [4, 5].

Anion	Donor number/kcal mol^{-1}
PF_6^-	-6.2
BOB^-	-3
BF_4^-	7.3
$TFSI^-$	7.6
ClO_4^-	7.6
FSI^-	15.5
OTF^-	20.4
NO_3^-	22.2
$DFOB^-$	-

Table S2. Donor number and dielectric constant of glyme-based solvents [6, 7].

Solvent	Donor number /kcal mol^{-1}	Dielectric constant	Melting point /°C	Viscosity (mPa·s)
G4	12±4	7.78	-30	3.3-3.7
G2	18±1	7.63	-64	0.98-1.0
DME	19±1	7.07	-58	0.42-0.46

Table S3. Parameters of the anode-free Al@C||NFM pouch cell.

**Parameters**	**Cell (at –40 °C)**
NFM percentage/%	95.5
Mass loading of NFM cathode/mg cm^{-2}	22 (active mass: 21)
Cathode size	4.5 × 9.0 cm^2
Anode size	4.8 × 9.4 cm^2
Operating temperature/°C	–40
Voltage cut-off/V	4.0
Discharge capacity/Ah	2.14
Energy density based on the active mass of cathode and anode/Wh kg^{-1}	358
Energy density based on the mass of entire cell/Wh kg^{-1}	204

REFERENCES

1. Lu T, Chen F. Multiwfn: A multifunctional wavefunction analyzer. *J Comput Chem*. 2012; **33**(5): 580-592. doi: 10.1002/jcc.22885
2. Humphrey W, Dalke A, Schulten K. VMD: Visual molecular dynamics. *J Mol Graph*. 1996; **14**(1): 33-38. doi: 10.1016/0263-7855(96)00018-5
3. Abraham MJ, Murtola T, Schulz R *et al.* GROMACS: High performance molecular simulations through multi-level parallelism from laptops to supercomputers. *SoftwareX*. 2015; **1-2**: 19-25. doi: 10.1016/j.softx.2015.06.001
4. Zhou P, Xiang Y, Liu K. Understanding and applying the donor number of electrolytes in lithium metal batteries. *Energy Environ Sci*. 2024; **17**(21): 8057-8077. doi: 10.1039/D4EE02989E
5. Schmeisser M, Illner P, Puchta R *et al.* Gutmann Donor and Acceptor Numbers for Ionic Liquids. *Chemistry – A European Journal*. 2012; **18**(35): 10969-10982. doi: doi.org/10.1002/chem.201200584
6. Lutz L, Yin W, Grimaud A *et al.* High Capacity Na–O2 Batteries: Key Parameters for Solution-Mediated Discharge. *J Phys Chem C*. 2016; **120**(36): 20068-20076. doi: 10.1021/acs.jpcc.6b07659
7. Di Lecce D, Marangon V, Jung H-G *et al.* Glyme-based electrolytes: suitable solutions for next-generation lithium batteries. *Green Chemistry*. 2022; **24**(3): 1021-1048. doi: 10.1039/D1GC03996B
